# Uniportal video-assisted thoracoscopic surgery esophagectomy outcomes in 40 consecutive patients

**DOI:** 10.1093/icvts/ivad034

**Published:** 2023-02-16

**Authors:** Sezer Aslan, Gamze Gul Tiryaki, Jeyhun Pashayev, Cagatay Cetinkaya, Ali Fuad Durusoy, Nezih Onur Ermerak, Hasan Fevzi Batirel

**Affiliations:** Department of Thoracic Surgery, Sirnak State Hospital, Sirnak, Turkey; Department of Thoracic Surgery, Marmara University School of Medicine, Istanbul, Turkey; Department of Thoracic Surgery, Marmara University School of Medicine, Istanbul, Turkey; Department of Thoracic Surgery, Uskudar University School of Medicine, Istanbul, Turkey; Department of Thoracic Surgery, Beykoz State Hospital, Istanbul, Turkey; Department of Thoracic Surgery, Marmara University School of Medicine, Istanbul, Turkey; Department of Thoracic Surgery, Biruni University School of Medicine, Istanbul, Turkey

**Keywords:** Uniportal VATS, Minimally invasive, Esophagectomy, Ivor Lewis, Intrathoracic anastomosis, Side-to-side anastomosis

## Abstract

**OBJECTIVES:**

Minimally invasive esophagectomy has improved over time becoming faster and less invasive. We have changed our technical approach from multiportal to uniportal video-assisted thoracoscopic surgery (VATS) esophagectomy over the years. In this study, we analysed our results with uniportal VATS esophagectomy technique.

**METHODS:**

This study was a retrospective analysis of 40 consecutive patients with the intent to perform uniportal VATS esophagectomy for esophageal cancer between July 2017 and August 2021. Demographic criteria, comorbidities, neoadjuvant therapy, intraoperative data, complications, length of stay, pathological data, 30- and 90-day mortality and 2-year survival data were recorded.

**RESULTS:**

Forty patients (21 female) were operated (median age 62.9 [53.5–70.25]). Eighteen patients (45%) received neoadjuvant chemoradiation. The chest part of all cases was started with uniportal VATS and 31 (77.5%) was completed uniportally (34 Ivor Lewis, 6 McKeown). The median thoracic operation time in minimally invasive Ivor Lewis esophagectomy was 90 min (77.5–100). The median time for uniportal side-to-side anastomosis was 12 min (11–16). Five (12.5%) patients had leak, and 4 were intrathoracic. Twenty-eight (70%) patients had squamous cell carcinoma, 11 adenocarcinoma and 1 squamous cell carcinoma with sarcomatoid differentiation. Thirty-seven (92.5%) patients had R0 resection. The mean number of lymph nodes dissected was 24 ± 9.5. Thirty- and ninety-day mortality was 2.5% (*n* = 1). The mean follow-up was 44 ± 2.8 months. Two-year survival was 80%.

**CONCLUSIONS:**

Uniportal VATS esophagectomy is a safe, fast and feasible alternative to other minimally invasive and open approaches. Comparable results to contemporary series are observed in perioperative and oncologic outcomes.

**Figure:**
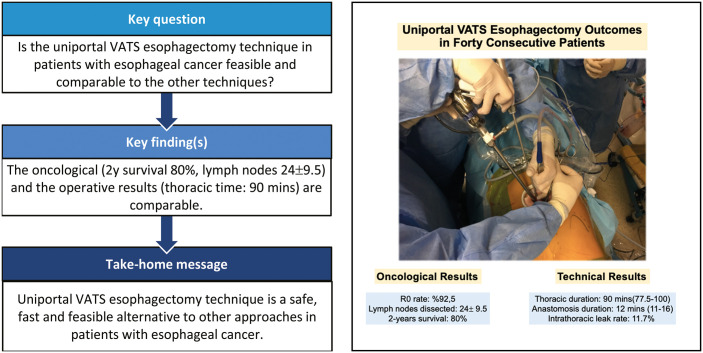

## INTRODUCTION

Esophageal cancer is the 8th common cancer and ranks 6th in cancer deaths [1]. Esophagectomy, as main surgical treatment, is a sophisticated surgical technique with high risk of morbidity and mortality. Minimally invasive techniques in esophagectomy have gained popularity in the last decade.

The surgical approaches in the esophagectomy are open and multiportal minimally invasive esophagectomy (MIE). Multiportal MIE leads to fewer pulmonary complications, shorter hospital stays, and less bleeding compared to open esophagectomy [2]. Surgical techniques are evolving with the goal of less invasiveness, smaller and fewer incisions. Uniportal video-assisted thoracoscopic surgery (VATS) technique is widely applied nowadays and is at least comparable in pain and morbidity to multiportal VATS for lung resection [3, 4]. There are limited data on the comparison of uniportal and multiportal VATS esophagectomy, but it would not be surprising to achieve similar results [5–9].

In the literature, uniportal VATS Ivor Lewis esophagectomy has been performed in small number of patients and these are only limited to case studies. Reproducibility and results are not available in a larger series yet. In this study, we aimed to test the feasibility of approach and report the outcomes.

## MATERIALS AND METHODS

### Ethics statement

Informed written consent was obtained from each patient and the study was approved by the Ethical Council of Marmara University Faculty of Medicine (09.2021.485; 6 December 2021).

### Study design

This study was a retrospective analysis of prospective database of 40 consecutive patients with the intent to perform uniportal VATS esophagectomy for esophageal cancer between July 2017 and August 2021. Demographic criteria, comorbidities, neoadjuvant therapy, intraoperative data, complications, length of stay, pathological data, 30- and 90-day mortality and 2-year survival data were recorded. Postoperative complications were graded according to Clavien-Dindo classification [10].

### Operative technique

#### Abdominal phase

**Figure 1:**
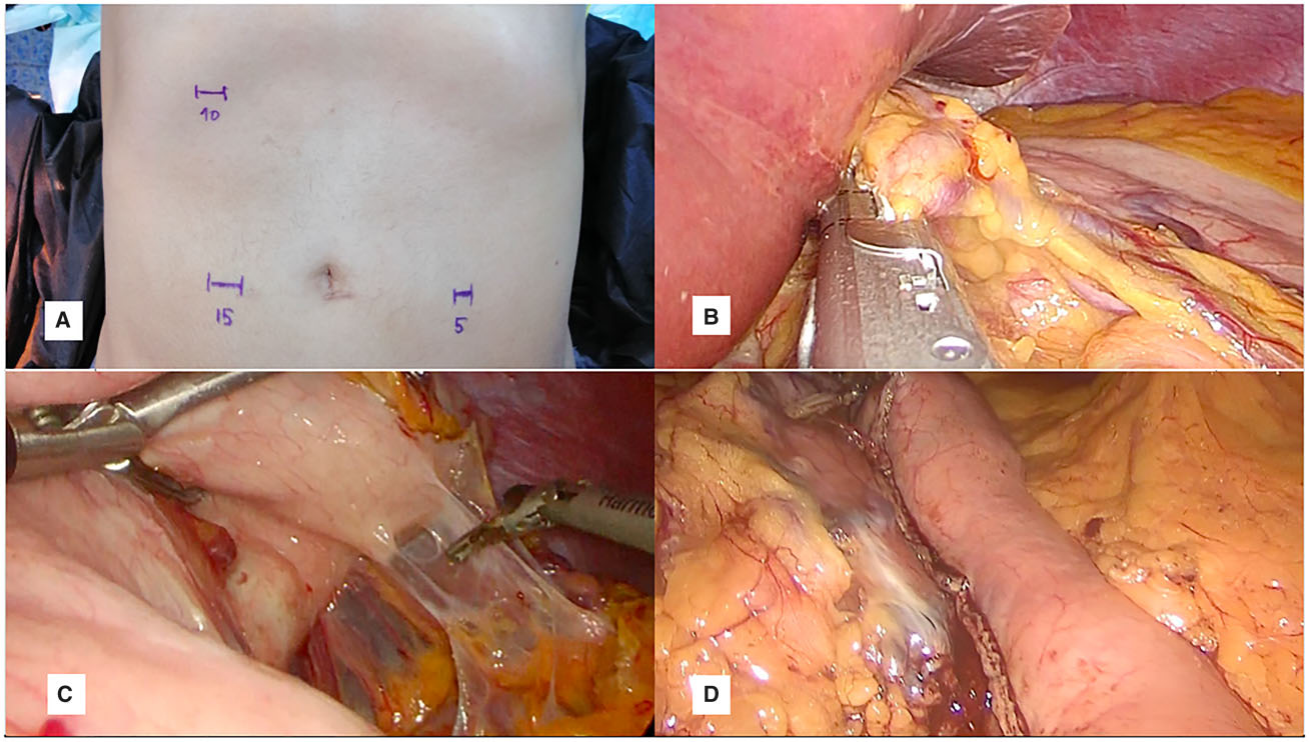
Abdominal phase. (**a**) Incision sites for three-port laparoscopy. (**b**) Division of left gastric vessels. (**c**) Non-grasping technique and open-jaw retraction of stomach. (**d**) Final view of conduit.

Three incisions were placed in the abdomen (Fig. 1a). A 12- to 15-mm laparoscopic port was placed 5-cm right lateral to the umbilicus. Second port (5 mm) was placed 5-cm left lateral to the umbilicus and the third port (10 mm) in the middle of right costal arch.

Right periumbilical port was used for the camera. A 10-mm Babcock clamp was introduced from the mid-costal port, and a 5-mm energy device from left periumbilical port. The operation started with dissection and division of the gastrohepatic ligament. The tissue was lifted with the 10-mm Babcock to the right side and this manoeuvre retracted the left lobe of the liver while exposing the left gastric area and hiatus.

Coeliac and left gastric lymph nodes with perihiatal fat were lifted to the specimen with clear visualization of left hepatic, splenic and left gastric arteries, crura and arcuate ligament. The left gastric artery and vein were prepared, and the camera was inserted from the mid-costal port. A 5-mm clamp lifted the left lobe of the liver through the left periumbilical port. A 30- to 45-mm endoscopic vascular stapler was inserted through the right periumbilical port and left gastric artery and vein were divided (Fig. 1b). In cases with small calibre left gastric vessels, vessel sealers were used. The right gastric and gastroepiploic artery were preserved in all cases.

Afterwards omentum was lifted with the Babcock clamp and gastrocolic ligament is visualized. Babcock clamp was used with an open-jaw manoeuvre without any grasping which allowed lifting the stomach with 1 jaw of the clamp and retracting it to the right side (Fig. 1c).

Endoscopic staplers were used through the right periumbilical port. The tip of the stapler was curved to the medial side after the first stapler firing and 3–5 firings were performed for gastric conduit formation (Fig. 1d).

We did not routinely apply pyloroplasty, feeding jejunostomy. Kocherization was decided according to the conduit length and level of anastomosis. Gastric stapler line was not oversewn. Hiatus enlargement was decided according to the intraoperative status of the hiatus. We gave utmost importance on the vascularity of stomach conduit and not to traumatize it with grasping or pulling.

#### Thoracic phase

**Figure 2:**
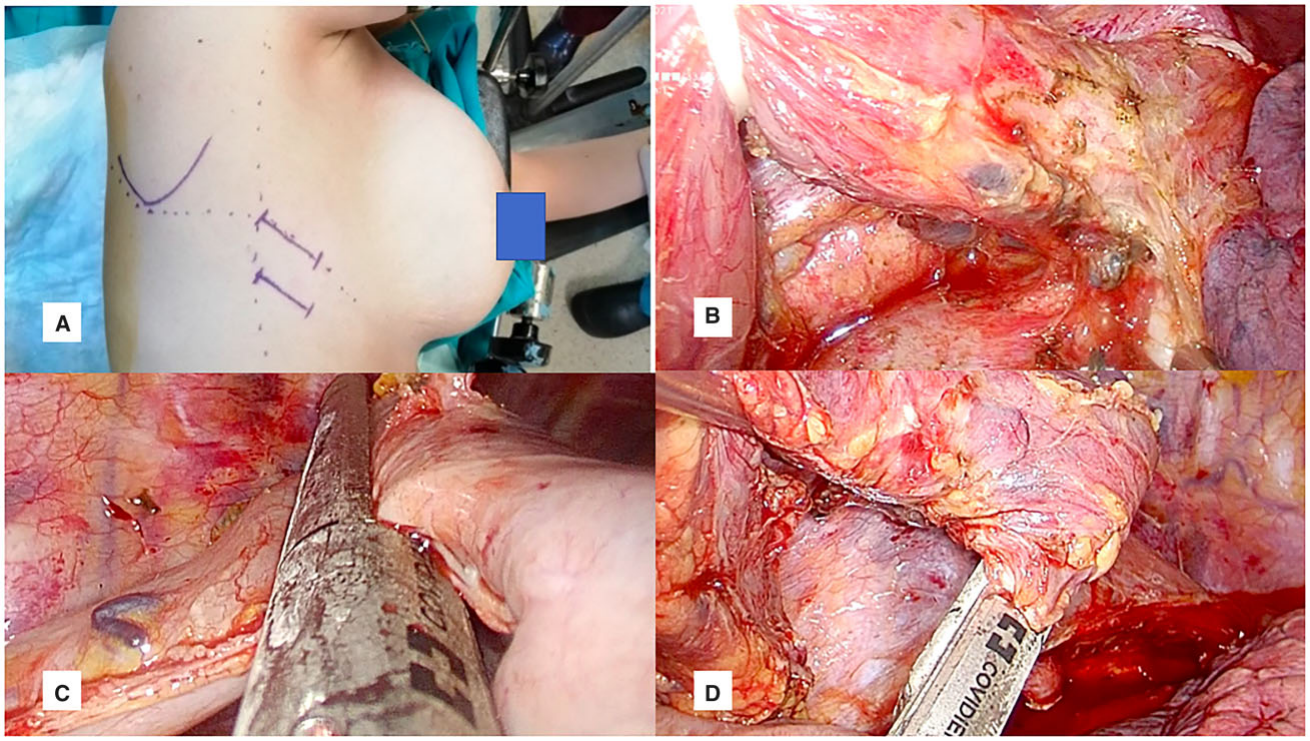
Thoracic phase. (**a**) Uniportal video-assisted thoracoscopic surgery incision at 5th or 6th intercostal space. (**b**) Encircled esophagus with penrose drain and exposure of subcarinal area. (**c**) Division of gastric conduit. (**d**) Division of esophagus.

The patients were evaluated for tracheobronchial system invasion with bronchoscopy following single lumen intubation in the operating room. After double lumen intubation, the patients were placed in the left lateral decubitus and tilted 30° anteriorly. A 4-cm incision was made at the 5th or 6th intercostal space on the posterior axillary line. Using the 6th intercostal space provided easy instrumentation in patients with small thoracic cavity (Fig. 2a). The lung was retracted anteriorly, and inferior pulmonary ligament was divided. Both posterior mediastinal and paravertebral pleura over the esophagus was opened till the azygos vein. The azygos vein was divided with a vascular stapler. Between the hemiazygos and esophagus dissection was continued deeply until the contralateral vagus nerve. Aortic branches were ligated with energy device. Esophagus was encircled with a penrose drain and lifted for retraction (Fig. 2b). Esophageal mobilization was completed with intrathoracic lymphadenectomy from diaphragmatic crus to the thoracic inlet. At this stage gastric conduit was pulled into the thorax with correct orientation during an Ivor Lewis procedure. When the planned level of intrathoracic anastomosis was determined, gastric conduit and esophageal ends were cut with endostaplers and the specimen was taken out (Fig. 2c and d).

### Intrathoracic anastomosis

**Figure 3:**
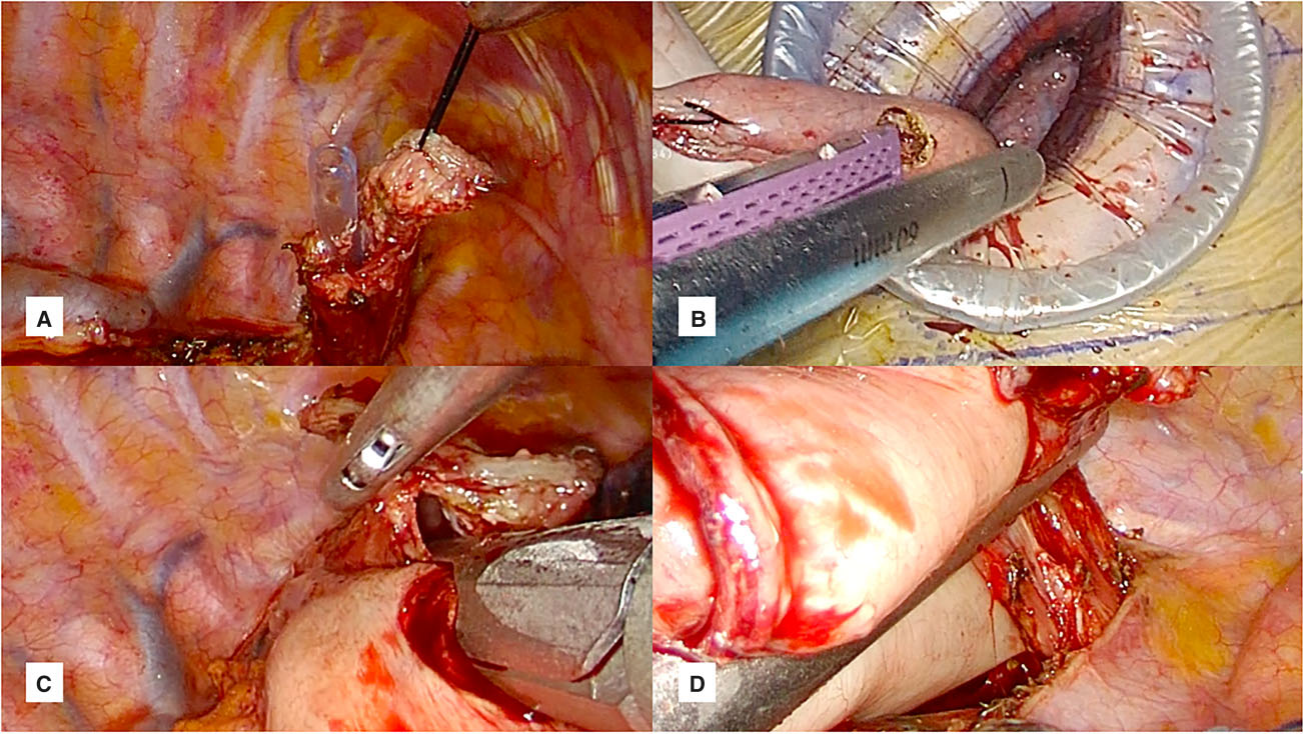
Side-to-side completely stapled anastomosis technique. (**a**) Gastrostomy opened outside the thorax and thick leg of stepler is placed. (**b**) Esophagostomy is opened, and nasogastric tube is advanced to guidance for stapler leg. (**c**) Both legs of stapler are advanced into the esophagostomy and gastrostomy to form the posterior wall of anastomosis. (**d**) Lateral wall of anastomosis is formed with 1 stapler firing and anastomosis is completed.

We preferred side-to-side completely stapled anastomosis in Uniportal VATS Ivor Lewis esophagectomy. The level of anastomosis was carefully measured to allow a tensionless approximation of stomach and esophagus. No 1 silk suture was placed on the esophageal tip close to the stapler line. Then, a small esophagostomy was opened at the esophageal end, and nasogastric tube was advanced to the chest cavity (Fig. 3a). The gastric conduit was pulled out of the incision in correct orientation and a small gastrostomy was opened. The thick leg of 60-mm tissue stapler was advanced in the gastrostomy and thin leg of stapler was placed in the esophageal opening taking the nasogastric tube as a guide (Fig. 3b). Posterior wall was formed with a single firing (Fig. 3c). It is important to ensure correct orientation of both structures and accurate tissue approximation at the same level during stapler firing. Then, both ends were retracted towards the lateral chest wall and the anastomosis was completed with firing of 1 or 2 60-mm staplers (Fig. 3d). A vertical linear stapler line was formed on the stomach and esophagus. Detailed uniportal VATS intrathoracic anastomosis technique is presented in Video 1.

### Statistical analysis

The data were evaluated using the SPSS 21 (Statistical Package for Social Sciences) statistical program. Frequency, percentage, standard deviation for mean and interquartile range for median values were used for demographic and perioperative data. Survival of the patients was calculated according to the date of death or last control. Kaplan–Meier was used for survival analysis.

## RESULTS

Forty patients (19 female) were operated during this period. The median age was 62.9 (53.5–70.25). Nine patients had major comorbidities (coronary artery disease with prior bypass in 3 patients, cardiomyopathy, cerebrovascular disease, chronic obstructive pulmonary disease). Twenty-five patients had at least 1 or more comorbidities (hypertension, diabetes, rheumatologic diseases in addition to major comorbidities). Eighteen patients (45%) received neoadjuvant chemoradiation, 2 of which were definitive. General characteristics of patients are shown in Table 1.

**Table 1:** General characteristics of patients

Variable	*n* (%)
Sex	
Male	19 (47.5)
Female	21 (42.5)
Age, median (IQR)	62.9 (53.5-70.25)
Comorbidities	
Hypertension	16 (40)
Diabetes mellitus	6 (15)
Coronary artery disease (3 with prior CABG)	5 (13)
Cardiomyopathy	1 (2.5)
Cerebrovascular disease	1 (2.5)
COPD	2 (5)
Pathology	
Adenocarcinoma	11 (27.5)
Squamous cell carcinoma	28 (70)
Squamous cell carcinoma with sarcomatoid differentiation	1 (2.5)
Neoadjuvant therapy	
Yes	18 (45)
No	22 (55)
Stage	
0	5 (12.5)
Ia	3 (7.5)
Ib	6 (15)
IIa	4 (10)
IIb	7 (17.5)
IIIa	4 (10)
IIIb	8 (25)
IVa	3 (7.5)
R status	
0	37 (92.5)
1	3 (7.5)
Lymph node dissected, mean ± SD	24 ± 9.5

CABG: coronary artery bypass grafting; COPD, chronic obstructive pulmonary disease; IQR: interquartile range; SD: standard deviation.

The thoracic part was started with uniportal VATS in all cases. Eight and one patients were converted to biportal and triportal VATS, respectively. Thirty-one (77,5%) were completed with uniportal VATS. There was no conversion to thoracotomy. Extra port incision was needed in 4 patients due to excessive pleural adhesions, in 1 patient due to difficulties in visualization of a large tumour, in 2 patients due to inappropriate angle of stapler for anastomosis and in 1 patient to support the anastomotic defect with sutures. Thirty-four patients had intrathoracic anastomosis and 26 of which were uniportal. Six patients underwent McKeown esophagostomy and had left cervical anastomosis. The median thoracic operation time in patients who underwent uniportal Ivor Lewis esophagostomy was 90 min (77.5–100). The median time for uniportal VATS side-to-side anastomosis was 12 min (11–16). The median blood loss was 75 ml (25–100). Operative data are shown in Table 2.

**Table 2:** Descriptive data of operative values and clinical outcomes

Variable	*N*	%
Thoracic approach		
Uniportal	31	77.5
Biportal	8	20
Triportal	1	2.5
Abdominal approach		
Triportal laparoscopy	37	92.5
Quadriportal laparoscopy	2	5
Laparotomy	1	2.5
Location of anastomosis		
Thorax	34	85
Neck	6	15
Abdominal operation time (min), median (IQR)	70 (57.5–85)	
Thoracic operation time (min), median (IQR)	90 (77.7–100)	
Intrathoracic anastomosis time (min), median (IQR)	12 (11–16)	
Total time, incision to closure (min), median (IQR)	180 (170–200)	
Length of hospital stay (days), median (IQR)	8 (7–10)	
Complications (Clavien-Dindo classification)		
2°	3	7.50
3°	8	20
4°	2	5
5°	1	2.50
Complications detailed		
Atrial fibrillation	3	7.5
Leak (intrathoracic cervical)	4–1	12.5
Chylothorax	3	7.5
Long-term Hiatal hernia	2	5
Pulmonary oedema	1	2.5
Intrathoracic leak	4	11.7
30- and 90-day mortality	1	2.5

Postoperative complications occurred in 14 patients. Grade 2 complications were seen in 3 patients, grade 3 in 8, grade 4 in 2 patients and grade 5 in 1. Five (12.5%) patients had leak and 4 of them were intrathoracic. Intrathoracic leaks were anastomotic (*n* = 2), due to conduit stapler line deficiency (*n* = 1) and conduit necrosis (*n* = 1). Thirty- and ninety-day mortalities were same (*n* = 1, 2.5%). The patient who died postoperatively underwent biportal VATS Ivor Lewis esophagostomy. Leak was due to the conduit stapler line necrosis. The patient had extensive pleural adhesions secondary to previous tuberculosis and moderate-to-severe chronic obstructive pulmonary disease [forced expiratory volume in 1 s: 1.7 l (54%), forced vital capacity: 3.04 l (74%), forced expiratory volume in 1 s/forced vital capacity: 55%]. Leak was detected on the first postoperative day and patient was reoperated. Repair was performed with primary sutures and serratus muscle flap. Leakage continued after revision. Necrosis was detected in >50% of the conduit and exclusion-diversion was performed. The patient died due to sepsis on the 5th postoperative day.

Twenty-eight (70%) patients had squamous cell carcinoma, 11 had adenocarcinoma and 1 had squamous cell carcinoma with sarcomatoid differentiation. Thirty-seven (92.5%) had R0 resection. Three patients had R1 resection and all of them was radial margin positivity due to margins closer than 0.1 mm in pathology specimens. The mean number of lymph nodes dissected was 24 ± 9.5. Detailed pathological results and staging are shown in Table 1. The mean follow-up was 44 ± 2.8 months and 2-year survival was 80%.

## DISCUSSION

Current expertise in thoracic surgery enables many surgeries to be performed through a single incision. This article reports one of the biggest series of uniportal VATS intrathoracic esophagostomy. Perioperative results and postoperative outcomes were like other MIE series. Major pulmonary and cardiac morbidity was 12.5%, and mortality was 2.5%. Oncologic adequacy of the resections (92.5% R0) was the same with open and MIE techniques.

The superiority of MIE over open techniques is proven. In a randomized controlled study published by Biere *et al.*, the data of 59 patients who underwent MIE in prone position were compared with 56 patients who underwent open esophagostomy. Less pneumonia (12–34%, *P* = 0.005), shorter hospital stays (11–14, *P* = 0.044) and less blood loss (200–475 ml, *P* < 0.001) were noted in the MIE group. In addition, there was no significant difference in parameters such as anastomotic leak, 30-day mortality, R0 resection and total number of lymph nodes removed. Open esophagostomy group was adversely affected in terms of pain (*P* = 0.002), talking (*P* = 0.008), general physical component (*P* = 0.007) and global health (*P* = 0.02) [11]. In a meta-analysis published by Akhtar *et al.* [12], less pulmonary complications, shorter hospital stays, and less blood loss were noted in the MIE compared with open technique. Oncologic outcomes were similar in the 3-year follow-up results of TIME trial by Straatman *et al.* [13]. There was no significant difference between the MIE and the open esophagostomy in terms of recurrence, disease free (*P* = 0.602) and overall survival (*P* = 0.633).

Surgical techniques have evolved to perform the same surgery with smaller incisions and less trauma. In the article published by Guo *et al.* [14], perioperative data (duration of surgery, amount of bleeding, leakage rates, length of hospital stay), oncological data (R0 status, lymph node counts dissected) and morbidity–mortality were compared and there was no statistically significant difference between types of MIE. Lee *et al.* published comparison of multiportal and uniportal MIE techniques. There was no difference in total surgery time (569–539 min, *P* = 0.239), length of hospital stays (26–20 days, *P* = 0.06) and total number of lymph nodes dissected (33–40, *P* = 0.06). When the pain scores after multiportal and uniportal VATS were examined, there was no difference in the postoperative 1st day (2.48–2.02, *P* = 0.053), but significantly less scores were observed on the postoperative 7th day in uniportal VATS (1.56–1.07, *P* = 0.001) [15]. Comparison of perioperative and pathological data with literature is shown in Tables 3 and 4. The reasons for relatively low neoadjuvant therapy rate compared to other series were patient refusal or pulmonary/cardiac comorbidities such as prior coronary artery by-pass grafting, dilated cardiomyopathy and tuberculosis scarring that might increase the risk of surgery after neoadjuvant treatment.

**Table 3:** Operative data compared with the literature

	Patients (*n*)	Technique	Location of anastomosis	Anastomotic technique	Thoracic time (min)^a^	Abdominal time (min)^a^	Total time (min)^a^	Blood loss (ml)^a^	Lymph node dissected^a^	Conversion to open (%)
Fabbi *et al.* [20]	36	Multiportal	Intrathoracic	Side to side semi-stapled	–	–	365 (240–480)	100 (50–1000)	24	–
Guo *et al.* [14]	41	Multiportal	Intrathoracic	Circular	–	–	268 ± 38	207 ± 74	18.6	1 (2.4)
Biere *et al.* [11]	59	Multiportal	Neck and Intrathoracic	Circular	–	–	329 (90–559)	200 (20–1200)	20	8 (14)
Nachira *et al.* [5]	12	Uniportal	Neck	Side to side	104 ± 20	–	–	–	10.4	0
Lee *et al.* [15]	16	Uniportal	Neck and Intrathoracic	Circular	–	–	608 ± 93	288 ± 361	30	1 (6.2)
White *et al.* [19]	170	Multiportal	Intrathoracic	Circular	–	–	391 (350–440)	250 (50–2500)	19	8 (4.7)
Luketich *et al.* [21]	1033	Multiportal	Neck and Intrathoracic	Circular	–	–	–	–	21	45 (4.5)
Current series, Aslan *et al.*	40	Uniportal	Neck and Intrathoracic	Side to side completely stapled	90 (77.7–100)	70 (57.5–85)	160 (150–180)	75 (25–150)	24	0

a Median values with inter quartile range or mean values with standard deviation.

**Table 4:** Pathological and postoperative morbidity data compared with the literature

	Patients (*n*)	Technique	Age^a^	Sex (M/F)	Pathologic diagnosis (adeno/SCC)	Neoadjuvant therapy (%)	R1 (%)	Length of stay (days)^a^	Anastomotic leak (%)	Pulmonary complications (%)	Cardiac complications (%)	30-Day mortality (%)
Fabbi *et al.* [20]	36	Multiportal	65 (29–83)	25/11	29/6	72	3 (8.3)	13 (7–64)	2 (5.6)	6 (16.7)	4 (11)	NS
Guo *et al.* [14]	41	Multiportal	60.5 ± 7	32/9	NS	NS	0	8.9 ± 4.7	2 (4.8)	NS	NS	NS
Biere *et al.* [11]	59	Multiportal	62 (34–75)	43/16	35/24	100	1 (2)	11 (7–81)	7 (12)	7 (12)	NS	1 (2)
Nachira *et al.* [5]	12	Uniportal	60.7 ± 8.6	10/2	5/6	58	0	15.7 ± 14.2	2 (16)	21 (6.7)	4 (33.3)	NS
Lee *et al.* [15]	16	Uniportal	58 ± 14.4	14/1	2/13	80	1 (6.2)	18.8 ± 7.7	2 (12)	0	NS	NS
White *et al.* [19]	170	Multiportal	64.7 (58–72)	147/23	160/10	84	NS	8 (8–10)	12 (7.1)	8 (4.7)	NS	1 (0.6)
Luketich *et al.* [21]	1033	Multiportal	64 (56–72)	807/226	727/105	31	8 (2)	8 (6–14)	49 (5)	85 (8.4)	50 (4.9)	17 (1.7)
Current series, Aslan *et al.*	40	Uniportal	63 (53–70)	19/21	11/29	45	3 (7.5)	8 (6–24)	4 (11.7)	3 (7.5)	2 (5)	1 (2.5)

a Median values with inter quartile range or mean values with standard deviation.

Adeno: adenocarcinoma; F: female; M: male; NS, not stated; SCC: squamous cell carcinoma.

Duration of surgery and perioperative data are important parameters to prefer a surgical technique. Nachira *et al.* [5] published uniportal VATS esophageal mobilization results of 12 patients. The mean duration of thoracic part was reported as 105 ± 21 min, and the mean lymph node count was 10.4 ± 3.9. In another study, Wang *et al.* [7] shared the results of 44 patients who underwent uniportal VATS esophageal mobilization. In that study, the mean duration of thorax was 163 ± 16 min and the mean number of lymph node dissected was 24 (14–36). The anastomosis duration was not included in these studies because neck anastomosis was preferred. In our patient group, the median uniportal VATS esophageal mobilization and anastomosis duration was 90 min and the mean number of lymph node dissected was 24 ± 9.5. The most important factor in shortening the surgical time was standardization of surgical steps such as dissection sequence and anastomotic technique [16].

Anastomotic leakage is one of the most important causes of surgical morbidity after esophagectomy. Sweet [17] mentioned that successful anastomosis can be achieved with no tension, atraumatic handling, separated sutures and good mucosal approximation. The most frequently used esophagogastric anastomosis technique is the circular stapler method. The technique involves placement of the anvil or orvil into the esophagus, then placing the stapler through the gastrostomy formed in the conduit and re-closing the gastrostomy after anastomosis [18]. In our patients, we preferred side-to-side completely stapled anastomosis. We avoided any trauma to the areas of anastomosis. The atraumatic approach starts at laparoscopy. The stomach is not grasped during mobilization. The stomach is retracted with movements such as pushing and lifting in appropriate positions. Anastomosis is made distal to the tip of the conduit and the tissue held by instruments while placing the staplers is removed after the anastomosis.

High intrathoracic anastomosis was preferred in Ivor Lewis procedure. So, there were no gastric emptying problems. In patients with leak, leak was controlled early on with stenting and patients were started with early oral feeding if there is no sign of leak. Thus, feeding jejunostomy were not needed in those patients.

When we analyse the reasons for leakage in our patients, tension was the reason in 1 patient. In another patient, preoperative uncontrolled hypertension was followed by a hypotensive episode which was most likely the reason of conduit hypoperfusion. Other leaks occurred in 2 patients with history of chemoradiation, advanced age (>75), hypertension and diabetes mellitus.

Performing procedures from a single incision in uniportal surgery has its own difficulties. These difficulties and the learning curve can be overcome with surgical experience. Surgical experience emerges as an important parameter for perioperative outcomes. In an article of 170 cases published by White *et al.*, patients were examined in 4 groups according to chronological order. According to the data, it is seen that there were no anastomotic leaks in the last 40 patients (*P* = 0.055) and this data came close to being statistically significant. In addition, significant improvement was observed in the last group in terms of length of stay and 90-day re-admission [19].

### Limitations

Our study has several limitations. Number of patients is limited, and we do not have a control group as the study was designed as a feasibility trial. It reflects the practice by a single surgical team and thus reproducibility will be tested over time following application of the technique by other groups. For such a feasibility trial we were able to achieve an 80% uniportal VATS completion rate.

## CONCLUSION

Uniportal VATS approach in esophageal cancer resection is a safe, fast and feasible alternative to other minimally invasive and open approaches in patients with esophageal cancer. Perioperative and oncologic outcomes are comparable to other approaches.

## Informed consent

Informed consent was obtained from all individual participants and the study was approved by the Ethical Council of Marmara University Faculty of Medicine (09.2021.485; 6 December 2021).

**Conflict of interest:** Dr Hasan Batirel is a consultant with Johnson and Johnson and receives fees and honoraria. Other authors have no conflict of interest to declare.

## Data Availability

All relevant data are within the article and the data underlying this article will be shared on reasonable request to the corresponding author.

## ABBREVIATIONS

MIE: Minimally ınvasive esophagectomy
VATS: Video-assisted thoracoscopic surgery
